# Reduced *rDNA* Copy Number Does Not Affect “Competitive” Chromosome Pairing in *XYY* Males of *Drosophila melanogaster*

**DOI:** 10.1534/g3.113.008730

**Published:** 2014-01-21

**Authors:** Keith A. Maggert

**Affiliations:** Department of Biology, Texas A&M University, College Station, Texas 77843-3258

**Keywords:** *Drosophila*, male meiotic/meiosis pairing, aneuploidy, *Y* chromosome, ribosomal DNA

## Abstract

The ribosomal DNA (*rDNA*) arrays are causal agents in *X-Y* chromosome pairing in meiosis I of *Drosophila* males. Despite broad variation in *X*-linked and *Y*-linked *rDNA* copy number, polymorphisms in regulatory/spacer sequences between rRNA genes, and variance in copy number of interrupting *R1* and *R2* retrotransposable elements, there is little evidence that different *rDNA* arrays affect pairing efficacy. I investigated whether induced *rDNA* copy number polymorphisms affect chromosome pairing in a “competitive” situation in which complex pairing configurations were possible using males with *XYY* constitution. Using a common normal *X* chromosome, one of two different full-length *Y* chromosomes, and a third chromosome from a series of otherwise-isogenic *rDNA* deletions, I detected no differences in *X-Y* or *Y-Y* pairing or chromosome segregation frequencies that could not be attributed to random variation alone. This work was performed in the context of an undergraduate teaching program at Texas A&M University, and I discuss the pedagogical utility of this and other such experiments.

Sex chromosome pairing in the heterogametic *Drosophila* male is mediated by the ribosomal DNA (*rDNA*) (McKee 1996, 2004), an array of tandem repeated *35S* pre-ribosomal RNA (rRNA) genes (Ritossa and Spiegelman 1965; Wellauer and Dawid 1977; Long *et al.* 1981b). Specifically, in meiosis I sequence repeats in the 240-bp intergenic “nontranscribed spacer” assure pairing and disjunction between the *X*-linked and *Y*-linked pre-rRNA transcription units (McKee and Karpen 1990; McKee *et al.* 1992; Merrill *et al.* 1992; Ren *et al.* 1997; McKee 2009). The causes and regulation of *rDNA* pairing remain areas of investigation; however, it is established that the minimum number of *rDNA* repeats to confer pairing between an *X* and *Y* is quite small (Appels and Hilliker 1982; McKee 1996).

The *rDNA* is highly polymorphic between populations, between individuals within populations, between cells within individuals, and between chromosomes within cells (Tartof 1973; Spear 1974; Long and Dawid 1980; Terracol and Prud’homme 1986; Lyckegaard and Clark 1989; Eickbush *et al.* 1997; Perez-Gonzalez and Eickbush 2002; Cohen *et al.* 2003; Perez-Gonzalez *et al.* 2003; Averbeck and Eickbush 2005; Stage and Eickbush 2007; Greil and Ahmad 2012). The copy number of rRNA cistrons is variable, ranging from tens to hundreds per array in most wild and laboratory strains. The spacer sequences between the transcription units are highly polymorphic, consisting of variable repeats of core elements that are thought to direct transcriptional enhancement, likely manifesting as varied levels of expression of each copy (Long *et al.* 1981a; Averbeck and Eickbush 2005; Stage and Eickbush 2007). The transcription units themselves may be interrupted by the *R1* and *R2* retrotransposable elements, and the presence of transcriptional and posttranscriptional regulatory mechanisms directed to these retroelements adds to the complex genetics of the *rDNA* (Perez-Gonzalez *et al.* 2003; Eickbush *et al.* 2008). The unusual arrangement of the *rDNA*—tandem expressed genes that alone account for approximately 50% of all nuclear transcription (Warner 1999)—renders them inherently unstable and prone to damage and loss (Peng and Karpen 2008, 2009; Guerrero and Maggert 2011). Finally, at least in some cases, there are special mechanisms to amplify or delete the *rDNA* genes in specific cells or at specific times of development (Ritossa 1968; Tartof 1973; Endow 1980, 1982; Hawley and Tartof 1985; Hawley and Marcus 1989). Consequently, the molecular-genetics of the *rDNA* has been refractory to simple approaches aimed at revealing cause–effect relationships. The location of the *rDNA* in heterochromatin in *Drosophila* has aggravated the difficulty in performing standard manipulative experiments by denying the use of many standard molecular-genetic tools.

The polymorphic *rDNA* array loci are located on the short arm of the *Y* chromosome and in the centric heterochromatin of the *X* chromosome, and are genetically redundant because either males or females can survive and accommodate all translational needs with only one *Y*-linked or *X*-linked *rDNA* array. Of the 100–600 copies found in natural and laboratory populations (Lyckegaard and Clark 1989), as few as 90 are sufficient for viability (although given the exceedingly complex regulation, an exact number is difficult to determine) (Terracol *et al.* 1990; Paredes and Maggert 2009a); the role of supernumerary copies is unclear, although their existence is apparently ubiquitous in eukaryotes (Long and Dawid 1980). Although significant *rDNA* copy number variation is found at either *X*-linked or *Y*-linked *rDNA* arrays, analyses of natural polymorphisms in *rDNA* gene copy number have not detected any quantitative chromosome segregation phenotype associated with *rRNA* gene copy number. Lyckegaard and Clark (1989) showed that *Y* chromosomes isolated from wild caught flies varied in *rDNA* copy number. They quantified the *rDNA* copy number on those chromosomes and correlated aneuploidy (loss and nondisjunction) with *rDNA* copy number, reasoning that *rDNA* copy number polymorphism, specifically low copy number, might result in chromosome pairing defects and loss or nondisjunction. They found no significant correlation between *X*<->*Y* disjunction in male meiosis and *rDNA* copy number (Clark 1987; Lyckegaard and Clark 1989). This is consistent with observations that very few *rDNA* copies—an order of magnitude fewer than found on natural Y chromosomes—are sufficient to assure pairing and disjunction (Appels and Hilliker 1982; McKee 1996, 1998). These real data are supported by my anecdote, years of using a *Y*-linked *rDNA* deletion series has never expressed an obviously high rate of nondisjunction (which would be readily apparent because of genetic markers on the *Y* chromosomes). However, in most experiments, and in my own observations using *Y* chromosomes with polymorphisms in *rDNA* copy number, *Y* pairing was not challenged by other pairing partners that could compete for the *X*-linked *rDNA* pairing sites. In that regard, it remains possible that faithful *X-Y* pairing is potentially reinforced by other pairing systems that act on *X* and *Y* after the homologous autosomes pair: even an unpopular boy may find a dance partner provided he is the last one available on the dance floor. Thus, it is conceivable that *rDNA* copy number polymorphisms confer a slight (or regulated) advantage that altered pairing arrangements would only be appreciable or detectable in “sensitized” situations, for instance, in a laboratory assay when multiple competing chromosome and pairing configurations are possible. Alternatively, the multiplicity of pairing sites in natural *rDNA* arrays may allow trivalent arrangements (Cooper 1964), obviating models of competition. Regardless, with three chromosomes moving to two poles, preferential co-orientation and segregation in *XYY* aneuploid males may reveal subtle quantitative effects of *rDNA* intergenic spacer sequences as pairing sites. Such hypothetical effects might have consequence in wild populations because male meiotic nondisjunction leads to *XYY* and *X0* males, and to *XXY* females, and might have repercussions in altering in sex ratio, inheritance of B or other supernumerary chromosomes, inheritance of heterochromatic sequence, and inheritance of *Y*-linked genes. Although *XYY* males and *XXY* females are not common in natural populations, they are not overly rare: the seminal study by Calvin Bridges showed that approximately 1 in 1000 males are *XYY* as a consequence of meiotic nondisjunction in females (from *XX* eggs fertilized by *Y*-bearing sperm, and the subsequent *XY*-bearing eggs fertilized again by *Y*-bearing sperm) (Bridges 1916a, 1916b). Bridges tested the pairing efficacy of chromosomes in *XYY* males and reasoned that both *Y* chromosomes were equally likely to pair with the *X*; however, he did not use individually marked chromosomes or chromosomes with *rDNA* copy number polymorphisms, and therefore he could determine a rate, but not whether *rDNA* copy number was salient.

Work by Grell (1958) and Lyttle (1981) both showed that different *Y* chromosomes differed in their pairing and disjunction in meiosis of *XYY* males; however, in both cases the data supported biases in progeny classes to be a result of postmeiotic embryonic inviability and not because of preference during pairing or segregation during meiosis. Neither set of work could ascribe any differences specifically to *rDNA* copy number. Few *rDNA* copies are sufficient to assure complete disjunction (Appels and Hilliker 1982), and even a single supernumerary copy is sufficient to alter segregation patterns based on *rDNA*-mediated pairing (Karpen *et al.* 1988; McKee and Karpen 1990; McKee 1996). My laboratory created and characterized a series of *rDNA* deficiencies from a common ancestor *Y* (Paredes and Maggert 2009a), allowing me to now test whether *rDNA* copy number reduction affected pairing of *X* and *Y* chromosomes in male meiosis of *XYY* aneuploids. I considered the *Y* chromosomes of this study to be isogenic at all loci except the *rDNA* based on fertility and cytology. After an initial period of *rDNA* magnification (Paredes and Maggert 2009a), the copy number has been robustly measured for years (J. Aldrich and K. Maggert, data not shown). I do not know the absolute number of *rDNA* genes on any of these chromosomes. Instead, I rely on fraction relative to amplification of a dispersed multicopy *tRNA* gene; however, the relative *rDNA* copy number that corresponds to the *bobbed-lethal* and *wild-type-bobbed* transitions of the deletion series is consistent with the wild-type *Y*,*10B* (see below) having approximately 300 total *rDNA* cistrons (Paredes and Maggert 2009a).

I used this *rDNA* deletion series to test pairing between an *X* chromosome, one of two “wild-type” (full-length) *Y* chromosomes, and the *Y* bearing a shortened *rDNA* array. Males of genotype *X*, *rDNA*^wild-type^/*Y*, *rDNA*^wild-type^/*Y*, *rDNA*^Deficiency^ were outcrossed and all progeny were scored so that the chromosome composition of the sperm could be inferred, as could the frequencies of different pairing arrangements in meiosis I. I show that pairing and segregation in males with three sex chromosomes are unperturbed by *rDNA* copy number polymorphisms on one *Y*, because the proportions of all resulting phenotypic classes were statistically indistinguishable regardless of the identity of the wild-type *Y* chromosome or the *rDNA* copy number on the supernumerary *Y* chromosome. This finding establishes that even when challenging the pairing of natural *X* and *Y* chromosomes, a third chromosome with copy number polymorphisms of the *rDNA* on the *Y* chromosome is unlikely to quantitatively affect pairing or segregation.

## Materials and Methods

### *Drosophila* husbandry

*Drosophila* cultures were kept on molasses-yeast-cornmeal food at 25° and 80% relative humidity. Crosses were performed with 3–5 females and 2–3 males per vial and cultured for 5 days before transferring or dumping; offspring were counted on days 14 and 18 after vials were set. Genotypes are described in *Results*, but published references are *Y*, *B* (Maggert and Golic 2005), *Y*, *ROMA* (Maggert and Golic 2002), and *Y*, *10B*, and derivatives (Paredes and Maggert 2009a, 2009b).

### Chromosome nomenclature

“*X*” is *X*, *y*^1^ *w*^67c23^. “*Y*, *B*^S^” is *B*^S^*Y*. “*Y*, *ROMA*” is *Y*, *P*{*y*^+mDintz^ *w*^BR.E.BR^ = *SUPor-P*}*ROMA*. These three are considered “wild-type” chromosomes in terms of *rDNA*. The chromosomes with manipulated *rDNA* copy number are derived from *y*^+^*Y*, *rDNA*^+^ *P*{*FRT*(*RS3*).*y*}*10B*, which is referred to as *Y*, *rDNA*^wt-10B^; by nature of it being the progenitor, it contains 100% *rDNA* by definition. Deficiency chromosomes are referred to as “*Y*, *rDNA*^Df^” (generally) or “*Y*, *rDNA*^Phenotype-Number^” (when denoting a specific chromosome; *bb* = *bobbed* and *l* = *lethal* when the *Y* is made the sole source of *rDNA*).

### DNA extractions and real-time PCR

DNA was extracted in a solution containing 100 mM Tris pH 8.0, 50 mM EDTA, and 1% SDS (added fresh). Flies were macerated using a Kontes pestle, proteinase K was added to 0.5 mg/mL, and the sample was incubated at 65° for 1 hr. Samples were then organic extracted four times with Tris-buffer phenol, phenol-chloroform-isoamyl alcohol (25:24:1), chloroform, and, finally, ethyl ether. DNA was ethanol-precipitated and resuspended in distilled water and normalized to 1 ng/µL. Real-time PCR was performed as described (Paredes and Maggert 2009a); primers were AGCCTGAGAAACGGCTACCA and AGCTGGGAGTGGGTAATTTACG for the *18S* rRNA and CTAGCTCAGTCGGTAGAGCATGA and CCAACGTGGGGCTCGAAC for *tRNA*^K-CTT^. Samples were run in triplicate (or more) for each sample of DNA, and DNA from females of genotype *C*(*1*)*DX*, *y*^1^ *f*^1^ *bb*^0^/*y*^+^*Y*, *rDNA*^+^ *P*{*FRT*(*RS3*).*y*}*10B*) was run on every separate PCR reaction plate to normalize between experiments.

### Statistical analyses

Regression functions and coefficients in Figure 3 were calculated using the CORREL, SLOPE, and INTERCEPT functions of Apple Numbers version 2.3. Data in Table 3 and Figure 4 were analyzed using Bayesian inference of 0.975 confidence intervals for the difference of means; exclusion of 0% from that interval was taken as a significant deviation between samples. Statements of lack of significant difference in sex ratios, differences between pooled and vial-separated progeny classes for each chromosome set, and *rDNA* copy number were all inferred the same way.

Statistical power (required N for specified P-value to discriminate differences in progeny classes) was calculated using the t distribution (and alpha = 0.05) using the average, SD, and N values from the data in Table 2.

## Results and Discussion

### Chromosome stocks used to test pairing and segregation are themselves without obvious nondisjunctional phenotypes

Males were generated with chromosome compositions *X*/*B*^S^*Y*/*Y*, *rDNA*^Deficiency^ or *X*/*Y*, *ROMA*/*Y*, *rDNA*^Deficiency^. Because single males were used to initiate all crosses in this set of experiments, the *Y*s were effectively isogenized; therefore, any polymorphisms that arose in the fly stocks since their establishment were removed for this set of experiments.

The *X* was isogenized in the laboratory and, as of 2005, the spontaneous nondisjunction rate in females was 0.06% (5 exceptional progeny of 8220 total progeny; C. Alfonso-Parra, unpublished data). This is consistent with the primary nondisjunction values of Bridges (1916a, 1916b), indicating that, at a minimum, there were no strong modifiers of female meiotic pairing in the genetic background. The “wild-type” *Y* chromosomes are from unrelated sources; *Y*, *B*^S^ and *Y*, *ROMA* were obtained from Kent Golic in 2001. The former is the original *B*^S^*Y* (Brosseau and Lindsley 1958), and the latter bears a variegating *SUPorP P*-element in cytological band h12 (Maggert and Golic 2002). Both are otherwise wild-type and cannot be separated by less than 100 years of independent variation (*Y*, *B*^S^ was originally from *y*^+^*Y*, which was generated in 1948; *Y*, *ROMA* was derived from an unmarked *Y* in approximately 2000). Stocks of both of these chromosomes showed normal meiotic nondisjunction (Table 1). The preponderance of *X0* progeny from *Y*, *ROMA* crosses highlights that I could not discriminate chromosome loss from meiotic nondisjunction using this class of progeny. Nonetheless, the rates of sex chromosome aneuploids in offspring should not unduly affect the analysis of sex chromosome pairing and segregation.

**Table 1:** Frequency of exceptional progeny from crosses between *X*, *y*^1^ *w*^67c23^ virgin females and males of genotype *X*, *y*^1^ *w*^67c23^/*Y* of the indicated identity

**Y Chromosome**	**Female**		**Male**		**% Female**	**% Aneuploid**
	**y w**	**B, y^+^w^+^, y^+^**	**y w**	**B, y^+^w^+^, y^+^**		
**B**	2228	9	10	1944	53.4	0.5
**ROMA**	942	0	4	1005	48.3	0.2
**10B**	3035	17	18	3020	50.1	0.6
**473**	969	3	13	958	50.0	0.8

Normal female progeny are expected to be yellow-bodied and white-eyed (“*y w*”), and normal male progeny are expected to be Bar (“B,” for the *Y*, *B*^S^ chromosome), yellow^+^ white^+^ (“y^+^w^+^,” for the *Y*, *ROMA* chromosome) or yellow^+^ (“y^+^,” for the *Y*, *rDNA*^wt-10B^ and *Y*, *rDNA*^l-473^ chromosomes). Primary exceptions (consequences of nondisjunction in meiosis of either males or females) are expected to be Bar, yellow^+^ white^+^, or yellow^+^ females and yellow white males. % Aneuploid was calculated from the sum of female and male exceptions; the latter class also includes chromosome loss events.

The data for spontaneous loss and nondisjunction of these chromosomes were collected from the same genetic background as the *X* above, further reinforcing the lack of high levels of meiotic nondisjunction in these flies. The progenitor *Y* chromosome with no *rDNA* deletion (chromosome *Y*, *rDNA*^wt-10B^) and the most extreme deletion recovered (*Y*, *rDNA*^l-473^) were tested for spontaneous loss or nondisjunction in the same way (Table 1); levels of neither *XXY* females nor *X0* males were elevated, and the sex ratios were statistically indistinguishable from random (50%) in every case. Each chromosome of the *rDNA* deletion series is reported as a fraction of the initial amount of *rDNA* before deletion (Tables 2 and 4) because I could not determine cardinal copy number (Paredes and Maggert 2009a).

**Table 2:** Frequency of each class of progeny from *XYY* males

**Y chromosome**			**rDNA**	**N**	**N**	**Average**	**SD**	**%**	**%**	**Pairing: L, M**		**Pairing: M, N**		**Pairing: L, N**	
**1**	**2**		**Size**	**Vials**	**Flies**	**Flies/Vial**	**Flies/Vial**	**Female**	**Aneuploid**	**X**	**12**	**X1**	**2**	**X2**	**1**
B	10B	Sum (total)	100%	32	3360	105	44	49.2	41.8	15.8%	8.4%	16.8%	23.4%	16.5%	19.1%
		Average (vials)						49.3	42.8	15.2%	8.7%	17.8%	22.4%	16.2%	19.6%
		SD (vials)						6.3	10.3	7.9%	4.9%	6.8%	8.8%	6.0%	7.7%
B	465	Sum (total)	83%	5	522	131	12	51.4	39.5	20.4%	8.5%	14.7%	28.0%	16.3%	12.1%
		Average (vials)						50.7	39.8	20.0%	9.1%	14.3%	27.8%	16.4%	12.5%
		SD (vials)						8.2	18.0	15.2%	4.8%	8.3%	9.8%	6.0%	6.6%
B	183	Sum (total)	76%	4	626	125	37	50.6	42.9	16.3%	8.6%	14.9%	21.1%	19.3%	19.7%
		Average (vials)						50.5	43.2	16.0%	8.7%	15.0%	21.1%	19.5%	19.8%
		SD (vials)						4.7	7.1	5.1%	3.1%	5.0%	3.9%	2.4%	2.9%
B	484	Sum (total)	55%	5	847	169	58	53.5	46.9	14.8%	8.1%	17.2%	18.4%	21.5%	20.0%
		Average (vials)						54.3	46.9	15.9%	8.5%	17.0%	18.0%	21.4%	19.2%
		SD (vials)						5.5	2.4	7.3%	1.7%	3.1%	2.1%	2.0%	5.0%
B	503	Sum (total)	52%	15	1219	81	31	52.1	41.5	19.0%	8.4%	14.4%	17.6%	18.6%	21.8%
		Average (vials)						52.4	43.2	18.1%	8.9%	15.7%	17.5%	18.6%	21.3%
		SD (vials)						4.8	8.7	7.0%	3.6%	6.7%	5.0%	5.6%	5.9%
B	473	Sum (total)	46%	25	2738	110	53	49.7	40.8	16.7%	7.8%	16.7%	24.4%	16.3%	18.0%
		Average (vials)						49.4	40.7	16.7%	8.0%	16.0%	24.2%	16.6%	18.4%
		SD (vials)						5.7	10.5	10.1%	3.5%	5.6%	6.1%	5.1%	6.7%
ROMA	10B	Sum (total)	100%	9	1349	150	78	50.9	47.1	12.9%	9.0%	22.4%	22.4%	15.6%	17.6%
		Average (vials)						52.0	48.3	12.4%	8.7%	25.1%	22.2%	14.5%	17.1%
		SD (vials)						5.0	9.6	5.6%	3.0%	7.4%	2.0%	5.1%	5.6%
ROMA	465	Sum (total)	83%	5	978	196	76	48.1	44.6	12.0%	8.5%	18.8%	23.8%	17.3%	19.6%
		average (vials)						47.2	44.0	11.9%	8.7%	18.5%	24.1%	16.8%	20.0%
		SD (vials)						3.8	4.5	0.7%	1.7%	2.5%	2.4%	2.7%	2.4%
ROMA	183	Sum (total)	76%	5	950	190	36	49.2	45.3	14.5%	10.6%	18.2%	23.4%	16.4%	16.8%
		Average (vials)						48.7	45.2	14.2%	10.7%	18.3%	23.4%	16.2%	17.3%
		SD (vials)						4.8	2.7	3.3%	1.6%	1.4%	3.4%	1.8%	4.9%
ROMA	484	Sum (total)	55%	5	1026	205	68	44.0	44.3	10.6%	11.0%	18.3%	25.6%	15.0%	19.4%
		Average (vials)						43.7	44.1	10.6%	11.0%	18.1%	25.6%	15.0%	19.7%
		SD (vials)						2.6	1.5	0.7%	1.7%	1.5%	2.1%	0.8%	1.7%
ROMA	473	Sum (total)	46%	10	1404	140	63	48.0	46.9	11.8%	10.6%	19.9%	23.7%	16.3%	17.7%
		Average (vials)						47.7	47.6	11.2%	11.1%	20.0%	24.1%	16.6%	17.1%
		SD (vials)						3.3	3.4	2.5%	2.5%	3.5%	4.0%	2.4%	2.8%

**Figure 1:**
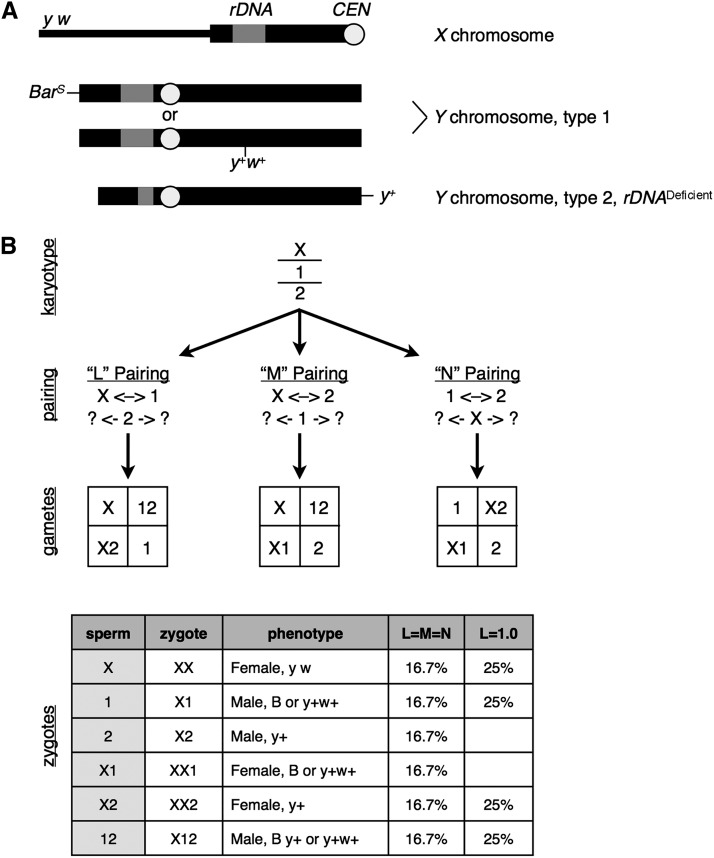
(A) Sex chromosomes used in this study. The *X* chromosome is mutant for *yellow* and *white* but has normal structure, including euchromatic arm (thin bar), pericentric heterochromatin (thick bar), the *rDNA* locus (gray), and a centromere (circle). In each cross there is a normal *Y* (“type 1”), either marked with *Bar* or a *P*-element containing *yellow^+^* and *white^+^* genes. The second type of *Y* (“type 2”) is marked with a *yellow^+^* gene and has a deletion of part of the *rDNA* array. (B) Sex chromosome aneuploid males (*XYY*, here the *Y* chromosomes are referred to by their types and are called “*1*” and “*2*“) can pair in three configurations. I denote “L” pairing to be between *X* and *1*, which assures disjunction of those two chromosomes, whereas *2* segregates at random, generating one of four possible sperm sex chromosome karyotypes: *X*, *1*&*2*, *X*&*2*, or *1*. “L” pairing and the other two pairing configurations (“M” and “N”) collectively produce six types of sperm (*X*, *1*, *2*, *X1*, *X2*, and *12*). Fertilization of an *X*-bearing egg will produce one of six types of zygote, and each can be separately identified based on dominant *Y*-linked marker genes. In the hypothetical case in which there is no preferred pairing between chromosomes (L=M=N”), the zygote genotypes will be equally frequent. In an extreme hypothetical case (“L=1.0” when “L” is the sole pairing type because *2* never pairs because of the defect in *rDNA*-mediated pairing), two progeny classes will be absent.

**Figure 2:**
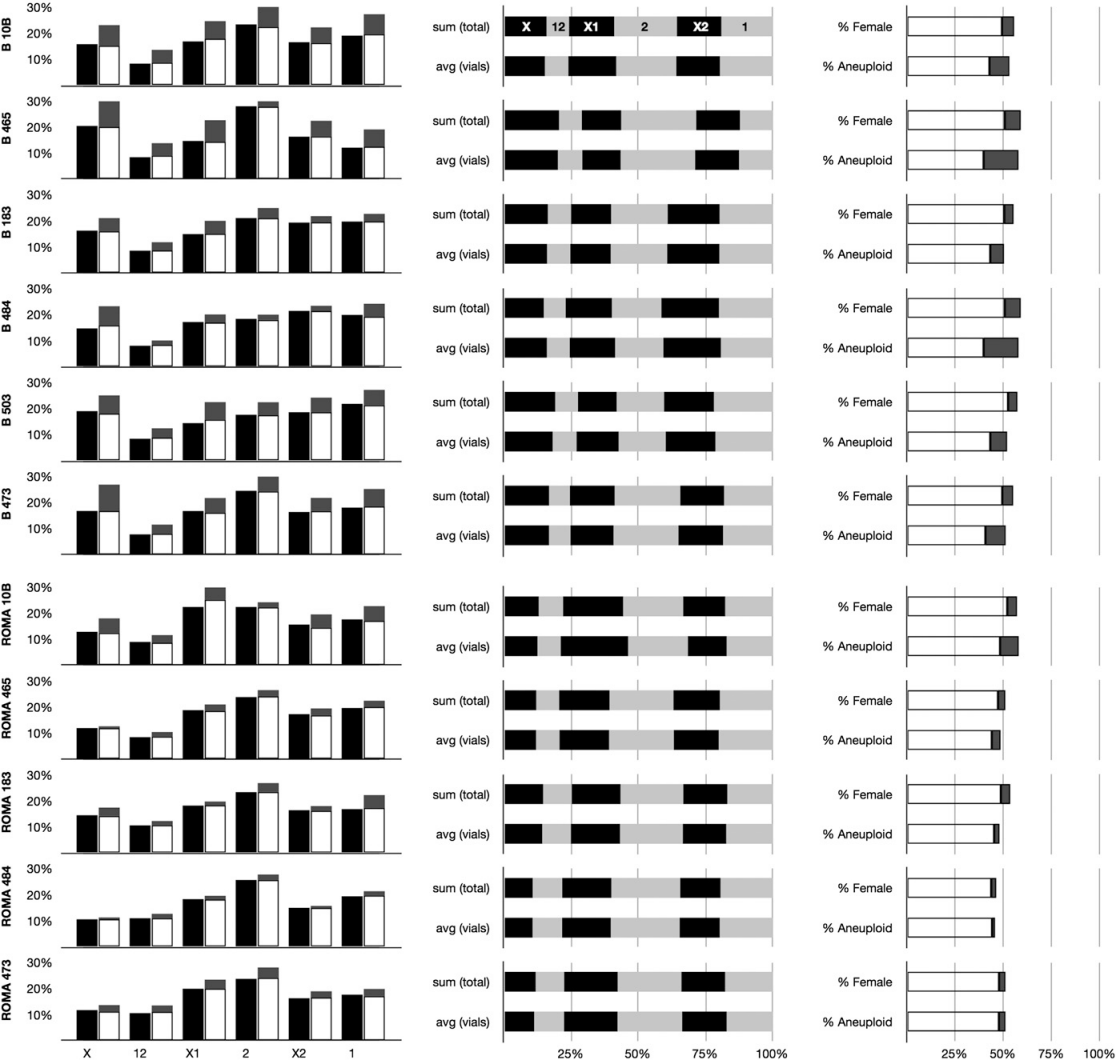
Graphical representation of segregation frequencies from Table 2, including pooled average (black) and averaged vials (white) with 0.67 confidence interval (based on average ± 1 SD) for each phenotypic class shown in gray. Graph column on the left shows progeny types separated for intragenotype comparison; graph column in the middle shows progeny types separated for intergenotype comparison. Graph column on the right shows averaged vial with 0.67 confidence intervals (±1 SD) for sex ratio and aneuploidy frequency. Sex ratio is normal, but subviability of aneuploid classes is evident for some crosses.

“Size” indicates *rDNA* copy number, relative to *Y*, *rDNA*^wt-10B^, which is defined as 100% (Table 4). Number of replicate vials and total flies are indicated [N (vials) and N (flies)], as are the average number of flies per vial (average) and SD of flies per vial. Sex ratio (% female) and fraction of sex chromosome aneuploids (% Aneuploid is a sum of *XXY* females and *XYY* males) are shown, as are progeny of each chromosome constitution. Pairing (L, M, N) refer to Figure 1A and indicated gamete types (*X*, *12*, *X1*, *2*, *X2*, *1*) refer to sperm karyotypes. Rows indicate values for all flies pooled into a single sum [sum(total)] and values for each separate vial averaged with SD [average(vial) and SD(vials)]. Pooled data are not outside the confidence interval derived from individual vials in any case. Data are shown graphically in Figure 2.

**Table 3:** Frequency of each class of progeny from vials separated by individual or by time

**Y Chromosome**			**X**		**12**		**X1**		**2**		**X2**		**1**	
**1**	**2**		**Average**	**SD**	**Average**	**SD**	**Average**	**SD**	**Average**	**SD**	**Average**	**SD**	**Average**	**SD**
**ROMA**	**10B**													
		**123**	0.123	0.020	0.092	0.028	0.225	0.003	0.203	0.040	0.182	0.010	0.223	0.020
		**1**	0.145	0.058	0.070	0.016	0.230	0.026	0.126	0.210	0.183	0.033	0.231	0.105
		**2**	0.119	0.012	0.128	0.039	0.226	0.042	0.149	0.112	0.166	0.058	0.205	0.056
		**3**	0.114	0.037	0.088	0.049	0.227	0.073	0.183	0.087	0.189	0.012	0.226	0.044
		**0.1**	0.103	0.019	0.065	0.026	0.256	0.051	0.147	0.116	**0.218**	**0.015**	0.205	0.058
		**0.2**	0.140	0.062	0.105	0.028	0.202	0.035	0.150	0.126	0.159	0.034	0.213	0.063
		**0.3**	0.135	0.018	0.116	0.056	0.225	0.043	0.155	0.179	**0.161**	**0.016**	0.245	0.090
**ROMA**	**473**													
		**123**	0.117	0.017	0.187	0.016	0.150	0.024	0.191	0.036	0.249	0.048	0.105	0.013
		**1**	0.140	0.048	0.164	0.064	0.158	0.010	0.236	0.042	0.188	0.059	0.113	0.044
		**2**	0.110	0.061	0.192	0.090	0.122	0.053	0.182	0.023	0.281	0.065	0.113	0.034
		**3**	0.112	0.059	0.190	0.050	0.169	0.056	0.157	0.026	0.282	0.078	0.089	0.022
		**0.1**	0.127	0.045	0.167	0.045	0.187	0.042	0.174	0.049	0.241	0.049	0.104	0.033
		**0.2**	0.095	0.046	0.216	0.068	0.137	0.029	0.194	0.007	0.246	0.070	0.112	0.019
		**0.3**	0.141	0.066	0.162	0.077	0.126	0.046	0.208	0.068	0.265	0.121	0.098	0.050

Two chromosome combinations were tested, *X*/*Y*, *ROMA*, *Y*, *rDNA*^wt-10B^, and *X*/*Y*, *ROMA*/*Y*, *rDNA*^l-473^. Each of three replicate vials (“1,” “2,” and “3”) from each genotype were transferred twice, establishing three temporal replicates (“0.1,” “0.2,” and “0.3”) from each. For example, 1.1, 1.2, and 1.3 were established by the same individuals, each offset by 5 days, whereas 1.1, 2.1, and 3.1 were all set on the same day with a separate set of parents. Progeny were scored independently and all nine of a genotype were considered as a set (“123”) or analyzed as progeny of parents or as progeny from a set time. In only one case do 0.975 confidence intervals of every pairwise comparison exceed 0 (bold), showing that within Bayesian limits there is no difference between progeny frequencies from any two vials, indicating that the variance seen is not attributable to differences in heritable or temporal factors.

**Table 4:** Copy numbers of *rDNA* arrays on chromosomes in this study

**Y Chromosome**	**N**	**Average**	**SEM**
B	11	112.3%	2.4%
ROMA	11	83.1%	1.7%
10B	Not applicable	100.0%	By definition
465	11	83.4%	1.5%
183	11	76.2%	2.2%
484	5	55.3%	4.2%
503	8	52.0%	5.8%
473	5	46.0%	11.3%

Real-time PCR was performed on progeny after the completion of the crosses show in Table 2 and Figure 2. All data are relative to *Y*, *rDNA*^wt-10B^. N is number of replicated real-time PCR reactions from a common pool of DNA purified from 40 sibling flies. All reactions were performed with reference (*Y*, *rDNA*^wt-10B^) reactions included, so SEMs of the reference were pooled into the *Y*, *ROMA*, *Y*, *B*, or *Y*, *rDNA*^Df^ data (Sokal and Rohlf 1995).

### Polymorphisms in *rDNA* copy number do not affect pairing or segregation in males

Analysis of progeny from *XYY* males is a complex but sensitive assay for the role of chromosome pairing because progeny will usually be derived from *X*-bearing eggs fertilized by *X*, *Y*, *XY*, or *YY* sperm, which are each derived from different pairing configurations (Figure 1). I considered three pairing configurations, which I termed L, M, and N for ease of discussion. L pairing is between the *X* and the first (wild-type) *Y* chromosome (henceforth described as “*1*,” *Y*, *B* or *Y*, *ROMA*), with the second *Y* chromosome (*Y* chromosome with the *rDNA* deletion under evaluation, henceforth described as “*2*”) segregating randomly. M is pairing between *X* and *2*, with *1* segregating randomly. N is pairing between the *Y* chromosomes, with the *X* segregating randomly. The “2+1” arrangements shown here represent an extreme case wherein two chromosomes show 100% pairing and disjunction (“2”), whereas the third chromosome (“+1”) moves randomly, as first envisioned by Bridges (1916a). Even if *XYY* aneuploid males do not exhibit 2+1 pairing, it is useful to model biases in pairing, co-orientation, segregation, and recovery of sex chromosomes that vary in *rDNA* copy number.

Identical sperm karyotypes can arise from two of the pairing configurations (*e.g.*, *12* sperm from L or M pairing, *X1* sperm from M or N pairing), so determination of whether *rDNA* copy number affects *X-Y* or *Y-Y* pairing requires evaluation of all six classes of progeny. The null hypothesis of random pairing and segregation predicts equal proportion of all progeny classes, whereas extreme bias (*e.g.*, exclusively L pairing) results in the absence of two classes.

Deviations in progeny classes may arise through other means but are expected to be inconsequential. Only very rarely would progeny be absent because of lethality, for instance, meiosis I or meiosis II nondisjunction in females producing nullo-*X* eggs fertilized by *Y* or *YY* sperm, or diplo-*X* eggs fertilized by *X* or *XY* sperm. These are expected to be a negligible minority of events (Table 1) and rely on female nondisjunction unaffected by differences in segregation in males, and thus are consistent across these experiments that manipulate sex chromosome ploidy in males. Similarly, chromosome loss (from nondisjunction or centromere/kinetochore dysfunction) could be readily detected by the use of marked *Y* chromosomes.

All progeny classes are unambiguously identifiable because the marker genes affect different aspects of development (eye color, eye shape, bristle color, body and wing color). *X1* and *X12* progeny, when the *Y* was *Y*, *ROMA*, could be discriminated because the *yellow*^+^ gene in *P*{*y*^+mDintz^ *w*^BR.E.BR^ = *SUPor-P*}*ROMA* is a partial gene, lacking the bristle enhancer element (Roseman *et al.* 1995), and is subject to position effect variegation (Roseman *et al.* 1995; Maggert and Golic 2002), whereas the *yellow^+^* gene on the *rDNA* deficiency series founder chromosome, *y*^+^*Y*, *rDNA*^Deficiency^ *P*{*FRT*(*RS3*).*y*}*10B*, is from a transposition between the *X* and *Y* (Maggert and Golic 2005), and thus is fully wild-type in expression. The *Y* chromosome in the stock from which the *XX* virgins were collected was unmarked, so that any nonvirgins involved in the cross would produce fertile *XY* yellow white male progeny and would be readily detectable; none were observed.

To obviate the possibility of genomic imprinting affecting *rDNA* activity and chromosome segregation, I created males with *X12* constitution by crossing *XX1* females to males bearing the *rDNA* deletion series. Males (*X12*) were then crossed to *XX* females, creating *X12* male progeny with a matroclinous *X* and two patroclinous *Y* chromosomes. Individuals were outcrossed to *XX* virgins and the progeny were scored on days 14 and 18. Each parental vial was transferred once or twice, creating two or three vials of progeny from the same parents. The data for the progeny are shown in Table 2 and are graphically represented in Figure 2.

The Tables report differences in offspring, which are indirectly results of differences in pairing. Three salient questions arise. First, do deficiencies of the *rDNA*, the known pairing centers of the *X* and *Y* in male meiosis I, affect chromosome pairing and segregation? Second, do *Y*, *Bar* and *Y*, *ROMA* chromosomes differ in their interactions with the *rDNA* deletion series? Third, are offspring recovered in expected (*i.e.*, Mendelian) frequencies? The answers to the first two questions, at least within the analytical limits of these statistics, are “no,” obviating any further analysis. The answer to the third question is “no,” although the subviability I saw was consistent regardless of the *Y* chromosome constitution and is consistent with previously described proportions in similar experiments (Grell 1958; Lyttle 1981).

My results indicate that while differences exist in the frequencies of each of the six progeny classes, *rDNA* copy number had no bearing on the frequencies of each class. By extension, *rDNA* copy number had no role in efficacy of meiotic pairing, segregation, centromere function, or viability.

Data are separately reported as a single pool of all flies collected from all vials [“sum (total)”] and as averages of individual vials [“average (vials)” and “SD (vials)”]. Comparisons of the former data with the latter show remarkable concordance, indicating that fluctuations in proportions of offspring classes seen in the individual vials were caused by random chance, as further explained below.

**Figure 3:**
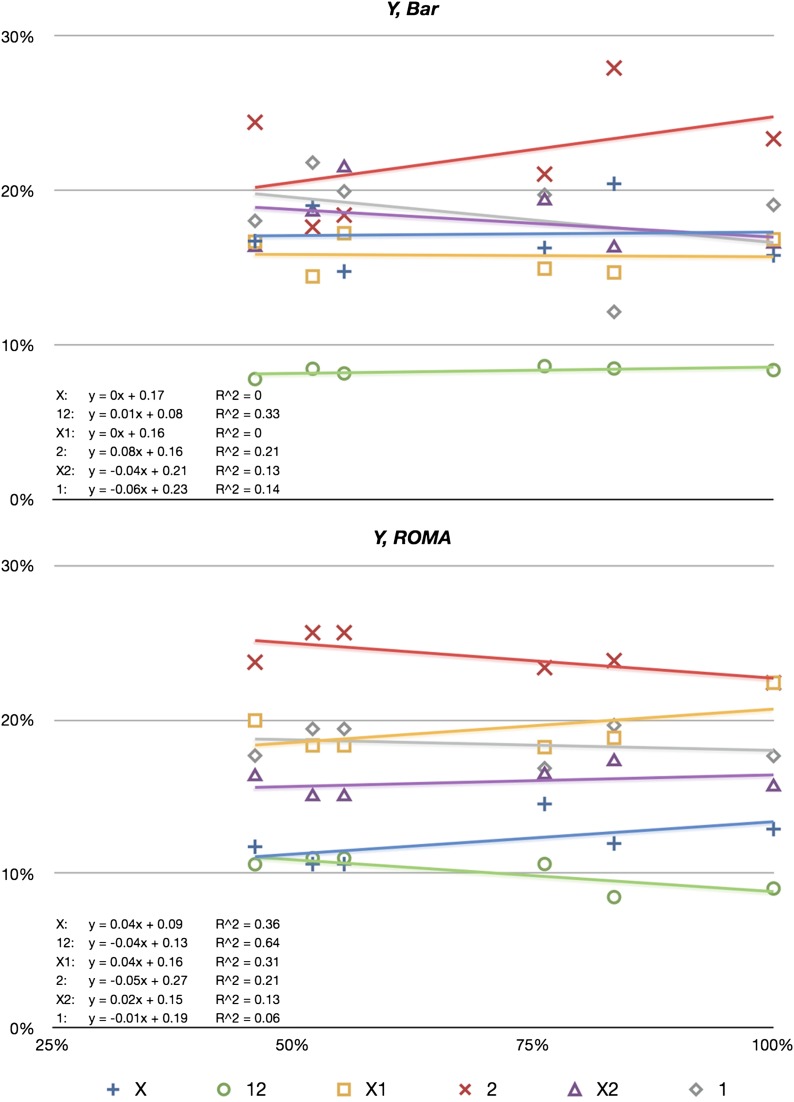
Monotonic regression for both *Y*#1 chromosomes as a function of *Y*#2 *rDNA* copy number. Formulae and R^2^ are shown for each phenotypic class. Data are from Table 4 (for *rDNA* copy number) and Table 2 (for class frequency).

Regression statistics were computed to determine if small effects could be divined (Figure 3). *rDNA* copy number was used as abscissa, and the ordinal values were from Table 2. In every case, both wild-type *Y*, *Bar* and *Y*, *ROMA* chromosomes, and every *rDNA* deletion chromosome, the slope was near zero, although the regression coefficients (R^2^) were widely disparate (from 0 to 0.64). Low R^2^ values indicate none of the observed trend could be attributed to *rDNA* copy number variation, and high R^2^ values ironically indicate a very high proportion of the trend could be attributed to a function with negligible input of *rDNA* copy number (because the slopes of the lines are all near zero).

The nonuniform frequencies of different progeny classes suggest that some pairing configurations may be preferred; however, it is not possible to determine if a particular pairing configuration is favored. The increased frequency of yellow white female offspring suggests that the *Y*s pair more frequently with each other than either does with the *X*. The corresponding classes of offspring (*XYY* males) are underrepresented, perhaps because of previously described subviability of *XYY* aneuploid males.

Meiotic drive of the sex chromosomes in male meiosis is an ideal way for a population to produce biased sex ratios in a population, and the particularities of sex determination in *Drosophila* (sex is determined by *X* dose rather than *Y* presence, and supernumerary *Y* chromosomes seem to have no phenotypic consequence in females) make control of *X-Y* pairing an appealing possibility for meiotic drive. However, the sex ratio in all of my experiments was the same and independent of *Y* chromosome constitution, strongly arguing against meiotic drive in *X12* males affecting either sex ratio in offspring or the overrepresentation of specific chromosomes.

Gynandromorphs or somatic mosaics attributable to *Y* chromosome loss were not seen in any of the experiments, arguing against pronounced mitotic instability or loss after fertilization. Concordantly, no flies were seen that could be interpreted as ultra-*Bar* or as two copies of *Y*, *ROMA*. Admittedly, the *Bar* and *white*^+^ markers could only be scored in the eyes, and the *yellow*^+^ of *Y*, *ROMA* could only be scored in the absence of the *rDNA* deficiency chromosomes. Because of the sectors of nuclei giving rise to epidermal anlagen in early embryogenesis, it is unlikely that any mitotic instabilities exist within the first three or four zygotic divisions.

### Random fluctuation and the limits of statistical power

The variance in Table 2 shows the SD, treating each vial as an independent subpopulation. Its high value indicates that variation between vials is broad, either as a consequence of meaningful differences in individuals or because of a large natural fluctuation in progeny types and phenotypic classes. If the latter is true, then a larger sample size would refine the SD, but not reduce it; therefore, it will do nothing to increase the likelihood of avoiding a type 2 error (inappropriate acceptance of a false null hypothesis). The correspondence between individual vials and grouped populations for each cross type, the consistency across all experiments, and isogeny between replicate vials all suggest that the differences in offspring classes are attributable to stochastic probability, arising from either pairing or other sources.

**Figure 4:**
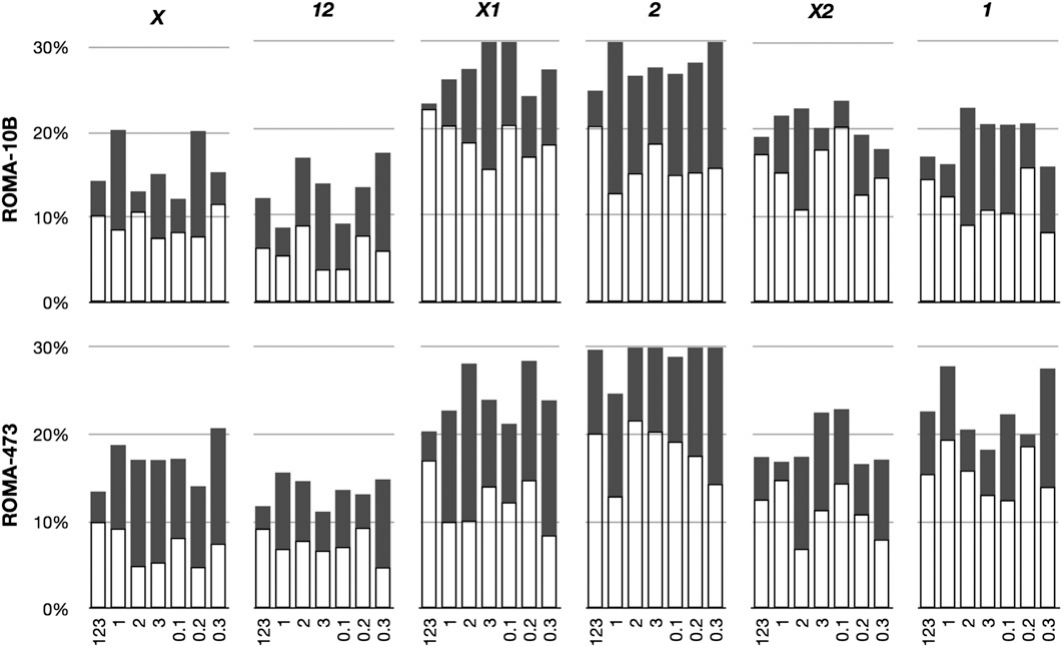
Graphical representation of segregation frequencies from Table 3. The 0.67 confidence intervals are shown in gray.

To address this assertion I separated data for individual crosses of *Y*, *ROMA* and *Y*, *rDNA*^wt-10B^ and of *Y*, *ROMA* and *Y*, *rDNA*^l-473^ into datasets corresponding to three replicate vials sired by separate individuals (vials “1,” “2,” and “3”) and all offspring sired by different fathers laid in the first, second, or third increments of 5 days (vials “0.1,” “0.2,” and “0.3”). These six separate populations were then compared to the collection of all nine vials (vial “123”), and the data for average and SD are shown in Table 3 and are shown graphically in Figure 4. Averages and SDs of individual parallel vials were not different from the same vial during three successive time periods, nor were they different from the assumption that all vials were independent, indicating that the entirety of the variance I detected in frequencies of offspring is random. The one exception (the first and third transfer vials of the *X2* class from the *Y*, *ROMA*/*Y*, *rDNA*^wt-10B^ cross) was significantly different (alpha = 0.025), but the lack of a corresponding difference in any other case suggests that this is not meaningful.

It is most clear from this analysis that complementary phenotypic classes are independent. For example, L pairing produces *X*, *12*, *X2*, and *1* gametes, thus *X* and *12* are complementary products, as are *X2* and *1*. If pairing was not dominated by randomness, then I expected complementary classes to co-vary; they did not, indicating that variation is random and under-representation is postmeiotic.

To determine the limits of my ability to resolve small effects on chromosome pairing and segregation by scoring final phenotypic classes, I calculated the required sample size for *X*/*Y*, *B*/*Y*, *rDNA*^wt-10B^ (which has the largest sample size) to resolve small differences in phenotypic class frequency. Assuming the measured sampling average was a true population distribution (µ), I calculated the N required to statistically distinguish each class from the cognate *X*/*Y*, *B*/*Y*, *rDNA*^l-473^ (the smallest *rDNA* array; hence, the one I expected would have the greatest impact on nondisjunction), with alpha = 0.05 and degrees of freedom set arbitrarily large (degrees of freedom = 1000). Using a normal t-distribution and hypothesis testing, I calculated that I would need a sample size of N = 275 vials to resolve the difference in frequencies of the *X*-bearing sperm phenotypic class, 266 for *12*, 90 for *X1*, 139 for *2*, 1374 for *X2*, and 286 for *1*. Note that these are the sample sizes required to establish a statistically significant difference in mean frequencies but, as noted above, no analysis can establish any consequence to differences in pairing effectiveness this small because random fluctuation is an order of magnitude greater than these differences in mean pairing frequency.

### Confirmation of *rDNA* copy number

At the conclusion of scoring, individual males bearing sole copies of the *Y*, *B*, *Y*, *ROMA*, and *Y*, *rDNA*^Deficiency^ series were outcrossed to virgin females of genotype *C*(*1*)*DX*, *y*^1^ *f*^1^ *bb*^0^/*Y*, *B*^S^, which possess no *rDNA* on their compound *X*-chromosomes. Female progeny were of genotype *C*(*1*)*DX*/*Y*, where the *Y* in question was isolated after the completion of the pairing/segregation assays. This had the benefit of assuring that any contaminating chromosomes that were inadvertently included in the experiments would be readily identifiable. For those chromosomes that do not contain sufficient *rDNA* for pupation and adult viability (*i.e.*, *Y*, *rDNA*^l-473^, *Y*, *rDNA*^l-503^, and *Y*, *rDNA*^l-484^), progeny were collected as larvae, along with *Y*, *rDNA*^wt-10B^ larvae for comparison.

DNA was extracted from progeny and used for quantitative real-time PCR as described. PCR reactions from crosses were performed to give quantification of *rDNA* copy number with pooled SEM. Data are presented in Table 4 and show *rDNA* copy number as a percentage of *Y*, *rDNA*^wt-10B^, which my laboratory has used as our wild-type standard in previous studies (Paredes and Maggert 2009b). All data are normalized to the *tRNA*^K-CTT^ gene as the internal standard. In no case did the *rDNA* copy number change significantly from the original published value.

### A note on the intersection of research and pedagogy

This work was initiated in the context of the first semester of the Capstone Research Program in Biology, a 1-hr class that met once per week during the spring semester of 2012 at Texas A&M University. The Capstone is a four-semester progression in the Department of Biology that targets sophomore biology majors to engage in active research, learn how biologists think, design experiments, perform trouble-shooting, collect data, assess its quality, analyze findings, and communicate the results to others. The first semester is specifically intended to foment interest in research, provide a modicum of exposure to the laboratory or field, and develop basic practical experience in designing and interpreting experiments. Semesters two and three provide a hybrid informatics-based course/laboratory research experience (which can be substituted by laboratory or fieldwork with a specific professor), and semester four teaches research communication.

In the semester one class, pilot crosses using *Y*, *rDNA*^wt-10B^, *Y*, *rDNA*^l-473^, and *Y*, *B*^S^ were set up and scored by the students, and the class analyzed the data as a group. The initial findings from that class were expanded later by me, but the essence of the experiment, including the salient acceptance of the null hypotheses discussed above, was performed by the students. This involved allowing small groups (3–4 students) to come into the laboratory to collect virgin flies, establish crosses, transfer vials, score phenotypes (*e.g.*, body color and sex), and count progeny. In the process of the experiment, the class had the opportunity to read selected research articles (Bridges 1916a; McKee 1996; Hempel 1966; Paredes and Maggert 2009a), discuss chromosome pairing and nondisjunction in general terms, create models and hypotheses for reasons why *rDNA* copy number might affect pairing, work through predictions for different efficiencies and establish alternative hypotheses, design and perform the crosses, analyze the vast amount of numerical data, decide on the best approaches (*e.g.*, pooling the data into one experiment or treat each vial individually), evaluate the strength of the data, and ultimately arrive at a conclusion.

For example, as a class we discussed every pairing configuration (L, M, and N), the meiotic segregation products, and sex determination by chromosome counting. This allowed the students to assure themselves that the sex ratio was expected to be 1:1 even in aneuploid crosses. It assured them that each of the six phenotypic classes could arise from multiple pairing configurations, and that every chromosome was equally likely to be present. These observations initiated discussion of expectations if L, M, and N were not equally likely, and under what conditions biases in phenotypic classes would be found.

The concrete connection between the theoretical discussion of pairing, seeing the *XYY* fathers, scoring the progeny to see the phenotypes, knowing exactly which flies had which chromosomes just by looking at them, and comparing expectations we had all agreed on with the actual data they collected were astoundingly effective. It was a significant advantage for students who were enrolled in Cell and Molecular Biology or in Genetics classes, because they could apply their classroom knowledge to living organisms. Even though the involvement in setting and counting the crosses was limited (approximately 15 min per student), every student reported feeling involved and having a vested interest in the discussions of chromosome segregation, the data collection, and even discussions of the benefits of Bayesian inference. Specifically, problems that arose during the experiment were easily used to launch discussions that they otherwise would not likely be exposed to in a normal science curriculum.

After the data were collected, the class observed (sometimes quite significant) deviation from 1:1 male:female ratios in individual vials. This fact gave the class a concrete example of variation with which they could easily associate because they had collected the data. It was an opportunity to discuss SD, probabilities of binomial distributions, simple statistics (*e.g.*, Student *t*-test, Bayesian confidence intervals, alpha *vs.* beta, and type I and II errors), and the propriety of pooling vials *vs.* averaging individual vials. I had more than one group count the same vials without their knowledge, which revealed experimenter error, allowing further discussion contrasting SEM with SD, and the intuitive logic of how to pool error into SE of the difference. Many (if not all) of these points are known to geneticists and are often highlighted in crosses or homework in sophomore-level genetics laboratories. The difference here was two-fold. First, the students were particularly engaged because this was “real” research in that it investigated a question that nobody had asked before. Thus, the result was not known beforehand. The realization that they were the first people in the history of humanity to see these data was a significant motivation. Second, the outcome was collaborative between all the students of the class, which fostered joint feelings of competition and caution. The value was profound, as exit interviews, course evaluations, and personal comments even more than 1 yr later have highlighted.

It was a boon to involve undergraduate students in this experiment, although it was difficult to align the joint considerations of low-cost, explicable biological phenomenon, accessible data collection, and synchronization of the organism’s life cycle with the weekly class session. Still, the ability to perform investigative science (as opposed to laboratory demonstration, however involved the students may be) was a positive outcome well worth the effort.
